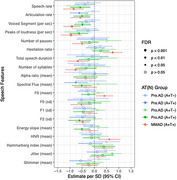# Effect of amyloid, tau and syndromic stages of Alzheimer's disease (AD) in speech markers: A potential scalable tool for screening in AD clinical trials

**DOI:** 10.1002/alz70856_102581

**Published:** 2025-12-26

**Authors:** Srinivasan Vairavan, Alankar Atreya, Nicholas Cummins, Claire L Lancaster, Jelena Curcic, Neva Coello, Anna‐Katharine Brem, Marion Mafham, Sarah Parish, Cornelia M Van Duijn, Gayle Wittenberg, Dag Aarsland, Chris Hinds

**Affiliations:** ^1^ Janssen Research and Development, Titusville, NJ, USA; ^2^ University of Oxford, Oxford, United Kingdom; ^3^ KCL, London, London, United Kingdom; ^4^ Novartis Biomedical Research, Basel, Switzerland; ^5^ Novartis Institutes for BioMedical Research, Basel, Switzerland; ^6^ King's College London, London, United Kingdom; ^7^ University of Oxford, Oxford, Oxford, United Kingdom; ^8^ University of Oxford, Oxford, Oxfordshire, United Kingdom; ^9^ Johnson & Johnson Innovative Medicine, Titusville, NJ, USA; ^10^ Kings College London, London, United Kingdom

## Abstract

**Background:**

Speech and language changes occur decades before the clinical diagnosis of Alzheimer's disease (AD). Digital tools for frequent speech assessments may have promise for early‐stage AD trials. Here, we present results from the RADAR‐AD study on the use of acoustic speech markers obtained from a story narration task, across AT(N) groups within the syndromic stages of AD.

**Method:**

Four study groups (Healthy controls (HC), preclinical AD (pre. AD), prodromal AD (pro. AD), and mild AD) were included in this cross‐sectional study of 8 weeks duration. The speech samples were collected using a voice‐based story narration task (Story Time), deployed within the Mezurio smartphone application. Analysis of speech from this task includes data from 159 participants across 12 different European languages (HC = 47, Pre.AD = 31, Pro.AD = 49, Mild AD = 32) collected across 6 different days during the study. To understand the effect of amyloid, tau and syndromic stages of AD on speech, we used a linear mixed‐effect model with participants, stories, and language as random effects and demographic variables as fixed effects and compared to A‐T‐ healthy controls.

**Result:**

For the preclinical subgroup, articulation rate was statically significant (FDR corrected) and was lower for A+T+ (β=‐0.62; *p* = .03). This feature was also lower for A+T+ in both prodromal (β=‐0.51; *p* =.03) and mild‐to‐moderate AD (β=‐0.58; *p* = .01), but not for the prodromal A+T‐ subgroups. The prodromal subgroups had statistically significant differences in jitter and voiced segments for the A+T‐ subgroup, and articulation rate, speech rate, peaks of loudness, voiced segments, and F2 frequency (sd) for A+T+.

**Conclusion:**

Our results suggest that subtle tau driven changes in speech fluency begin as early as preclinical stage highlighting the potential utilization of speech markers in improving screening in AD clinical trials.

This work has received support from the EU/EFPIA Innovative Medicines Initiative Joint Undertaking (grant No 806999). www.imi.europa.eu. This communication reflects the views of the RADAR‐AD consortium and neither IMI nor the European Union and EFPIA are liable for any use that may be made of the information contained herein.